# Glycemic Control, Inflammatory Mediators, and Periodontal Health: A Cross-Sectional Study in Patients with Diabetes

**DOI:** 10.3390/jcm14082847

**Published:** 2025-04-21

**Authors:** Vanessa Bolchis, Daniela Jumanca, Ramona Dumitrescu, Octavia Balean, Nicoleta A. Toderas, Simona Popescu, Anca Marcu, Catalin Marian, Atena Galuscan

**Affiliations:** 1 Translational and Experimental Clinical Research Centre in Oral Health, Department of Preventive, Community Dentistry and Oral Health, “Victor Babes” University of Medicine and Pharmacy, 300040 Timisoara, Romania; vanessa.bolchis@umft.ro (V.B.); jumanca.daniela@umft.ro (D.J.); galuscan.atena@umft.ro (A.G.); 2 Clinic of Preventive, Community Dentistry and Oral Health, “Victor Babes” University of Medicine and Pharmacy, Eftimie Murgu Sq. No 2, 300041 Timisoara, Romania; 3 Specialization in Clinical Psychology and Psychotherapy, Department of Psychology, Faculty of Sociology and Psychology, West University of Timișoara, 300223 Timisoara, Romania; nicoleta.toderas01@e-uvt.ro; 4 Second Department of Internal Medicine, “Victor Babes” University of Medicine and Pharmacy, 300041 Timisoara, Romania; popescu.simona@umft.ro; 5 Department of Diabetes, “Pius Brinzeu” Emergency Hospital, 300723 Timisoara, Romania; 6 Department of Biochemistry and Pharmacology, “Victor Babes” University of Medicine and Pharmacy, PtaEfimie Murgu Nr. 2, 300041 Timisoara, Romania; marcu.anca@umft.ro (A.M.); cmarian@umft.ro (C.M.)

**Keywords:** diabetes mellitus, periodontal disease, inflammatory markers, oral health awareness

## Abstract

**Background/Objectives**: The bidirectional relationship between diabetes mellitus (DM) and periodontal disease (PD) has garnered increasing attention due to shared inflammatory mechanisms and mutual disease exacerbation. In Romania, despite a high prevalence of diabetes and PD, integration of oral health into diabetes care remains limited. This study aimed to investigate the association between glycemic control, salivary inflammatory biomarkers (IL-1β, IL-6, MMP-8), and periodontal status in diabetic patients. Additionally, it evaluated patients’ awareness of oral health risks and their communication with healthcare providers regarding periodontal care. **Methods**: A cross-sectional, observational study was conducted between May and December 2024, involving 79 adult patients with confirmed type 1 or type 2 DM. Periodontal examinations assessed probing pocket depth (PPD), clinical attachment level (CAL), plaque index (PI), and bleeding on probing (BOP). Salivary samples were collected to quantify IL-1β, IL-6, and MMP-8. Participants also completed a structured questionnaire on oral symptoms, hygiene practices, and awareness of the diabetes–periodontitis link. Correlation and *t*-test analyses were used to explore associations between clinical, biochemical, and self-reported variables. **Results**: Most participants had advanced periodontitis (65.8% Stage IV; 72.2% Grade C). IL-1β and IL-6 were positively correlated (r = 0.34, *p* < 0.01), while MMP-8 correlated with PI (r = 0.28) and BOP (r = 0.26). Inflammatory markers showed weak correlation with HbA1c. Notably, patients with higher oral health knowledge reported worse clinical indices, suggesting increased symptom awareness rather than preventive effectiveness. **Conclusions**: This study reinforces the inflammatory link between DM and PD and highlights the need for integrated care models. Periodontal screening and education should be embedded within diabetes management, particularly in high-risk populations.

## 1. Introduction

Over the past few decades, the global prevalence of diabetes mellitus (DM) and that of periodontal disease have risen at an alarming rate, posing significant public health challenges [1,2,3]. Periodontal disease, a chronic inflammatory condition affecting the supporting structures of the teeth, is characterized by gingival bleeding, periodontal pocket formation, connective tissue breakdown, alveolar bone resorption, and ultimately, tooth loss. Beyond its local effects, accumulating experimental and clinical evidence suggests that periodontitis is intricately linked to the pathogenesis and progression of diabetes and its associated complications [4]. Conversely, diabetes is known to exacerbate periodontal disease by impairing immune responses, increasing inflammatory mediators, and altering host-microbial interactions [5,6]. This bidirectional relationship establishes a vicious cycle in which the presence of one condition aggravates the severity of the other, making their interplay an important area of research [7].

Diabetes mellitus affects approximately 415 million adults worldwide, with projections indicating an increase to 642 million by 2040. Type 2 diabetes mellitus (T2DM) accounts for most cases, while type 1 diabetes mellitus (T1DM) represents about 10%. Similarly, periodontal disease (PD) is highly prevalent, affecting 47.2% of adults over 30 years old and rising to 70.1% in individuals aged 65 and older. It is more common in men (56.4%) than in women (38.4%) and disproportionately affects individuals with lower socioeconomic status and smokers. The increasing retention of natural teeth in aging populations further contributes to the burden of periodontal disease, as prolonged exposure to microbial biofilms and inflammatory responses exacerbates tissue destruction over time [8]. Given the high prevalence of both conditions, it is essential to examine the underlying inflammatory mechanisms that drive their progression and mutual exacerbation, providing a foundation for targeted interventions.

In Romania, the rising prevalence of diabetes mellitus coincides with insufficient integration of oral health into chronic disease management. Previous studies [3,9] conducted on Romanian cohorts have shown that a substantial proportion of diabetic patients report symptoms associated with periodontal disease—such as gingival bleeding, halitosis, and tooth mobility—yet few receive routine periodontal evaluations or targeted oral hygiene guidance. Interdental cleaning practices remain uncommon, and awareness of the bidirectional relationship between diabetes and periodontal disease is limited among both patients and healthcare professionals. These trends are further compounded by structural gaps in dental care delivery. A multinational survey [10] revealed that Romanian general dentists display low confidence in periodontal risk assessment, with a mean knowledge score of 5.8 out of 9, and only 11.6% reporting access to a periodontist in their practice. Moreover, less than 40% of practitioners consistently offer oral hygiene instruction to all patients. Collectively, these findings emphasize the need for interdisciplinary collaboration, enhanced professional training, and preventive strategies focused on periodontal screening within diabetic care programs.

A growing body of evidence suggests that chronic hyperglycemia in diabetic patients contributes to an exaggerated inflammatory response in periodontal tissues, leading to increased production of pro-inflammatory cytokines and matrix metalloproteinases (MMPs), which drive tissue degradation and alveolar bone loss. Elevated levels of key inflammatory mediators, such as interleukin-1 beta (IL-1β), interleukin-6 (IL-6), and matrix metalloproteinase-8 (MMP-8), have been observed in the gingival crevicular fluid and serum of individuals with both diabetes and periodontitis, reinforcing the hypothesis of a localized inflammatory pathway that further exacerbates periodontal destruction [11].

The interplay between chronic hyperglycemia and periodontal inflammation highlights the multifactorial nature of periodontitis, where both microbial dysbiosis and dysregulated immune responses contribute to progressive tissue destruction. Periodontal disease is a multifactorial inflammatory condition characterized by a dysregulated host immune response to microbial biofilm, leading to progressive destruction of the periodontal ligament and alveolar bone. While bacterial pathogens initiate the disease, the severity and progression are largely determined by the host’s inflammatory response. Elevated levels of pro-inflammatory cytokines, such as IL-1β and IL-6, along with matrix metalloproteinases like MMP-8, contribute to the degradation of connective tissue and bone resorption, driving the clinical manifestations of periodontitis [12,13].

Moreover, poor glycemic control has been associated with delayed wound healing and increased susceptibility to infections, further complicating periodontal disease progression in diabetic individuals [14]. Epidemiological studies indicate that patients with poorly controlled diabetes exhibit a two- to threefold higher risk of developing severe periodontitis compared to those with well-regulated glycemia [15]. Conversely, periodontal disease itself has been implicated in worsening metabolic control, potentially contributing to insulin resistance through sustained low-grade inflammation. This interplay underscores the need for a more detailed investigation into the molecular mechanisms linking glycated hemoglobin and periodontal inflammation, with a focus on local mediators that may serve as potential biomarkers for disease progression [16].

Traditional treatments, such as scaling and root planning, primarily target bacterial biofilm but do not directly modulate the inflammatory response, which is crucial in disease progression and recurrence [17,18]. Investigating the role of key inflammatory mediators in periodontal tissue breakdown may provide valuable insights into disease pathogenesis and aid in identifying biomarkers for early diagnosis and targeted therapeutic approaches [19].

Given the complex interaction between diabetes mellitus and periodontal disease, the inflammatory mediators involved play a crucial role in disease progression and tissue destruction. Among them, interleukin-1 beta (IL-1β), interleukin-6 (IL-6), and matrix metalloproteinase-8 (MMP-8) are particularly significant due to their direct impact on periodontal tissue homeostasis. IL-1β is a potent pro-inflammatory cytokine that amplifies the immune response by promoting leukocyte infiltration and stimulating osteoclast differentiation, leading to alveolar bone resorption and periodontal tissue degradation [8,20]. Elevated levels of IL-1β in gingival crevicular fluid and saliva are strongly associated with increased periodontal disease severity, particularly in diabetic individuals, where hyperglycemia further enhances its production, prolonging inflammatory activity. Similarly, IL-6 plays a key role in immune regulation by stimulating osteoclastogenesis and contributing to soft tissue breakdown. Increased IL-6 levels have been linked to more severe periodontal destruction, especially in individuals with poor glycemic control, indicating a synergistic effect between diabetes and periodontal disease. MMP-8, an enzyme predominantly secreted by neutrophils, is responsible for collagen degradation within the extracellular matrix, contributing to periodontal ligament breakdown and CAL. Studies have shown that higher MMP-8 concentrations correlate with increased pocket depth and tissue destruction, making it a valuable biomarker for periodontal disease activity. The persistent elevation of these inflammatory mediators in periodontitis, particularly in the presence of diabetes, underscores their potential as key targets for therapeutic intervention aimed at mitigating periodontal inflammation and limiting disease progression [21].

Although the diagnostic and pathogenic relevance of IL-1β, IL-6, and MMP-8 has been increasingly documented, their integration into routine clinical risk assessment for diabetic patients remains limited. Saliva, as a non-invasive diagnostic fluid, offers a practical and patient-friendly medium for biomarker-based periodontal monitoring. However, standardized protocols for sampling, interpretation, and clinical application are still lacking, representing a significant gap between experimental evidence and real-world implementation. Further research is needed to validate threshold values, assess longitudinal changes, and explore how salivary cytokine profiling might enhance individualized risk stratification and early intervention strategies for diabetic patients with periodontal disease [22].

Despite the increased risk of periodontal complications in individuals with diabetes, awareness of this association remains limited. Many diabetic patients report experiencing symptoms such as gingival bleeding, tooth mobility, and dental sensitivity, yet few recognize these as potential complications of their condition. Additionally, discussions about oral health with diabetologists are infrequent, and preventive dental care is often overlooked. Understanding patient perceptions and behaviors regarding oral health is essential to identifying gaps in knowledge and improving preventive strategies in this high-risk population [23]. The simultaneous analysis of IL-1β, IL-6, and MMP-8—cytokines central to innate immunity and extracellular matrix degradation—provides a robust framework for evaluating inflammatory status. Previous literature suggests their additive value in capturing both local tissue destruction and systemic immune activation, which are key components of periodontal disease progression in diabetic patients.

This study aims to evaluate the association between glycemic control, inflammatory markers (IL-1β, IL-6, MMP-8), and periodontal health in patients with type 1 and type 2 diabetes. Additionally, it seeks to assess patient awareness of oral health risks, their experiences with symptoms such as gingival bleeding, tooth mobility, and dental sensitivity, and the extent to which they have received oral health guidance from their diabetologists. By analyzing correlations between periodontal parameters (PPD, CAL, PI, BOP), inflammatory marker levels, and glycemic control, this research aims to identify potential links between periodontal inflammation and metabolic dysregulation in diabetic patients. The findings may contribute to a better understanding of the inflammatory mechanisms involved and highlight the importance of early periodontal assessment, patient education, and interdisciplinary collaboration in managing diabetes-related oral health complications.

## 2. Materials and Methods

### 2.1. Study Design

This cross-sectional, observational, and correlational study was conducted between May and December 2024 at the Outpatient Diabetes Care Facility of the Pius Brînzeu County Emergency Hospital in Timișoara, Romania. After receiving their medical consultation, patients were referred for periodontal examination at the Faculty of Dental Medicine, Victor Babeș University of Medicine and Pharmacy, Timișoara. The study protocol was reviewed and approved by the university ethics committee, and all participants provided written informed consent before inclusion in the study. All procedures were carried out in accordance with the ethical standards of the institutional research committee and the principles outlined in the Declaration of Helsinki, under ethics approval number No. 05/30.01.2024.

### 2.2. Inclusion and Exclusion Criteria

The study included 79 patients who met the eligibility criteria. Participants were initially referred by their diabetologist to the Faculty of Dental Medicine, Victor Babeș University of Medicine and Pharmacy, Timișoara, for a comprehensive periodontal evaluation. To be included in the study, patients had to be 18 years or older and have a confirmed diagnosis of type 1 or type 2 diabetes mellitus. Following the periodontal assessment, only those diagnosed with periodontal disease were included in the study. Patients were excluded if they were found to have no signs of periodontal disease, if they were unable to provide informed consent due to psychological or cognitive impairments, if they declined participation, or if they were pregnant. Additionally, individuals who had received systemic antibiotic therapy or anti-inflammatory treatment within the past three months were excluded, as such treatments could influence inflammatory marker levels.

### 2.3. Data Collection

Each participant completed a standardized questionnaire designed to assess oral health symptoms, perceptions, and awareness regarding the link between diabetes and periodontal disease. The questionnaire gathered information on gingival bleeding during tooth brushing, tooth sensitivity or pain, tooth mobility, awareness of the diabetes-periodontal disease relationship, previous discussions with their diabetologist about oral health risks, past or present periodontal problems, and special oral hygiene recommendations received from their dentist.

In addition to the questionnaire, saliva samples were collected from each participant to quantify inflammatory markers, including interleukin-1 beta (IL-1β), interleukin-6 (IL-6), and matrix metalloproteinase-8 (MMP-8). A comprehensive periodontal examination was performed by resident dentists specializing in general dentistry under the supervision of experienced faculty members. The periodontal evaluation followed standardized guidelines, and data were recorded in the periodontal chart online (periodontal chart, Department of Periodontology, School of Dental Medicine, University of Bern, Switzerland) which included key clinical parameters such as periodontal disease staging and grading, PPD, CAL, plaque index (PI), and bleeding on probing (BOP).

To complement clinical and subjective data with molecular profiling, unstimulated whole saliva samples were collected under standardized conditions. Participants were instructed to abstain from eating, drinking (except water), smoking, and performing oral hygiene procedures for at least one hour prior to sample collection. All samples were obtained between 8:00 and 10:00 a.m. to minimize diurnal variation in salivary cytokine levels. Following collection, saliva samples were immediately placed on ice and transported for storage at −80 °C until laboratory analysis. These pre-analytical measures were implemented to preserve the stability of protein biomarkers and minimize variability in cytokine quantification across samples.

Salivary concentrations of IL-1β, IL-6, and MMP-8 were quantified using commercially available enzyme-linked immunosorbent assay (ELISA) kits (R&D Systems, Minneapolis, MN, USA). The assays were performed according to the manufacturer’s instructions, including duplicate measurements and standard curve generation for each biomarker. This methodology has been previously validated for use in salivary diagnostics of periodontal disease and is recognized for its sensitivity and reproducibility in measuring cytokine levels in oral fluids [24].

### 2.4. Periodontal Examination and Clinical Parameters

Periodontal assessments were conducted using the Community Periodontal Index of Treatment Needs (CPITN) probe, a specialized periodontal probe designed for standardized periodontal evaluation. This probe features a 0.5 mm ball tip to minimize trauma and improve patient comfort, along with calibrated markings at 3.5 mm, 5.5 mm, 8.5 mm, and 11.5 mm to ensure precise measurements of PPD. The lightweight design of the CPITN probe allowed for controlled probing force, enhancing the accuracy and reliability of periodontal measurements. Periodontal parameters were assessed at six sites per tooth, and all findings were systematically documented according to the Berne Periodontal Chart.

The clinical periodontal evaluation included periodontal staging and grading, along with detailed measurement of PPD, CAL, plaque index (PI), and bleeding on probing (BOP). These parameters provided an objective assessment of periodontal disease severity and its association with diabetes-related inflammatory changes.

### 2.5. Statistical Analysis

All statistical analyses were conducted using IBM SPSS Statistics, Version 23 (IBM Corp., Armonk, NY, USA). Descriptive statistics were used to summarize demographic, clinical, and questionnaire-based data, with continuous variables reported as means and standard deviations (mean ± SD), and categorical variables as frequencies and percentages.

Given the cross-sectional and observational design of the study, without a control group or pre–post intervention comparison, independent-samples *t*-tests were applied to assess differences between subgroups defined by self-reported oral health perception and gender. Due to the limited sample sizes within some categories, ANOVA procedures were not deemed appropriate.

To explore associations between periodontal parameters, salivary inflammatory biomarkers, glycemic control (HbA1c), and self-assessment scores, Pearson correlation analyses were performed. The statistical approach focused on identifying overall patterns of association across the cohort, rather than testing predefined group differences within periodontal classifications. A two-tailed *p*-value of ≤0.05 was considered indicative of statistical significance for all analyses.

## 3. Results

The analysis revealed relevant clinical and biochemical patterns reflecting the relationship between periodontal status, inflammatory response, and glycemic control in patients with diabetes mellitus. Results are structured to describe the participants’ demographic and clinical characteristics, periodontal condition, salivary concentrations of IL-1β, IL-6, and MMP-8, and the associations between these variables. In addition, findings related to oral health awareness and self-reported periodontal symptoms are presented.

### 3.1. Demographic and Clinical Characteristics of Participants

The study sample consisted of 79 adult patients previously diagnosed with diabetes mellitus. The mean age of the participants was 61.20 years (±SD), and the gender distribution indicated a modest predominance of female patients, with 55.7% (*n* = 44) being women and 44.3% (*n* = 35) men. Most participants (86.1%, *n* = 68) had type 2 diabetes mellitus, while a smaller proportion (13.9%, *n* = 11) were diagnosed with type 1 diabetes.

**Figure 1:**
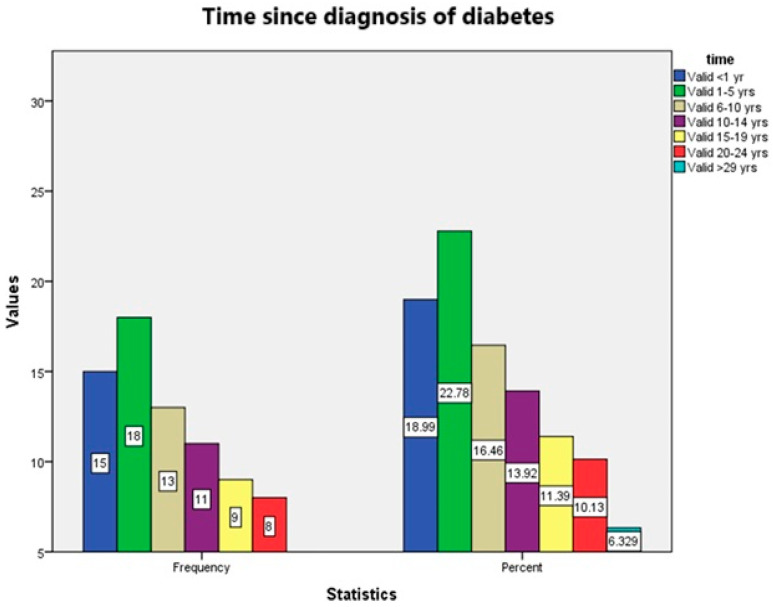
Frequency and percentage distribution of patients based on the duration of diabetes mellitus since diagnosis (*n* = 79).

In terms of disease duration, 22.8% of the participants reported a diabetes history of 1 to 5 years, followed by 19.0% who had been diagnosed within the last year. Additional subgroups included 16.5% with 6 to 10 years since diagnosis and 13.9% with 10 to 14 years. Fewer participants reported long-standing disease, with 16.4% indicating durations beyond 20 years. The distribution of diabetes duration is illustrated in Figure 1, showing a tendency toward a shorter time since diagnosis in this cohort.

The demographic profile of the sample reflects the typical epidemiological pattern of diabetes mellitus, with a higher proportion of type 2 diabetes and an age range suggestive of mid-to-late adulthood onset. These characteristics are summarized in Table 1 and provide a relevant context for interpreting periodontal and systemic inflammatory parameters in subsequent analyses.

### 3.2. Periodontal Parameters in Diabetic Patients

Clinical periodontal assessment revealed substantial soft tissue inflammation and loss of attachment among diabetic patients. The mean PPD was 4.00 mm (±0.69), and the mean CAL was –2.59 mm (±4.50), indicative of periodontal tissue breakdown. Inflammatory burden was also reflected by a mean bleeding on probing (BOP) of 55.14% (±18.93), and a mean plaque index (PI) of 45.73% (±20.27), highlighting generally insufficient oral hygiene practices.

These values reflect a considerable degree of periodontal tissue damage, emphasizing the clinical need for early periodontal screening and intervention in diabetic populations.

Based on clinical staging criteria, 65.8% of patients were diagnosed with Stage IV periodontitis, while 34.2% were diagnosed with Stage III. In terms of disease progression, 72.2% of the study group were classified as Grade C, indicating a rapid progression pattern, and 27.8% as Grade B. These data support the presence of moderate to advanced periodontal disease in this diabetic cohort. A summary of clinical periodontal parameters and classifications is presented in Table 2.

### 3.3. Correlations Between Inflammatory Biomarkers and Clinical Periodontal Parameters

Correlation analysis was performed using Pearson’s r coefficient to explore associations between salivary inflammatory markers and clinical periodontal variables. A significant positive correlation was observed between IL-1β and IL-6 (r = 0.34, *p* < 0.01), indicating that higher IL-1β levels were associated with increased IL-6 concentrations. The effect size was small (r^2^ = 0.11). In contrast, IL-1β was negatively correlated with MMP-8 (r = −0.24, *p* < 0.05) and with periodontal stage diagnosis (r = −0.23, *p* < 0.05), suggesting that higher IL-1β levels were associated with lower MMP-8 expression and lower disease stage; both associations showed small effect sizes (r^2^ = 0.05).

Similarly, IL-6 also showed a significant negative correlation with periodontal stage (r = −0.33, *p* < 0.05), with a small effect size (r^2^ = 0.11). MMP-8 demonstrated positive correlations with both bleeding on probing percentage (r = 0.26, *p* < 0.05) and plaque index percentage (r = 0.28, *p* < 0.05), again with small effect sizes (r^2^ = 0.07 for both).

These findings suggest modest but meaningful relationships between the local inflammatory profile and clinical measures of periodontal health in diabetic patients (Table 3).

Notably, inflammatory markers showed limited correlation with glycated hemoglobin (HbA1c), suggesting that local periodontal inflammation may operate partially independently of systemic metabolic control, a hypothesis explored further in the next section.

Although the observed correlations are modest in strength, they underscore the complex interplay between systemic inflammatory responses and local periodontal conditions in individuals with diabetes mellitus.

**Figure 2:**
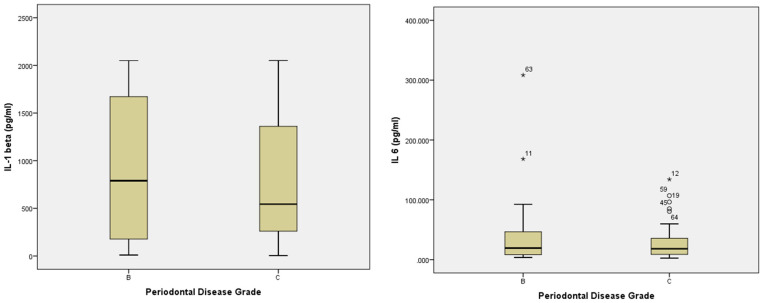

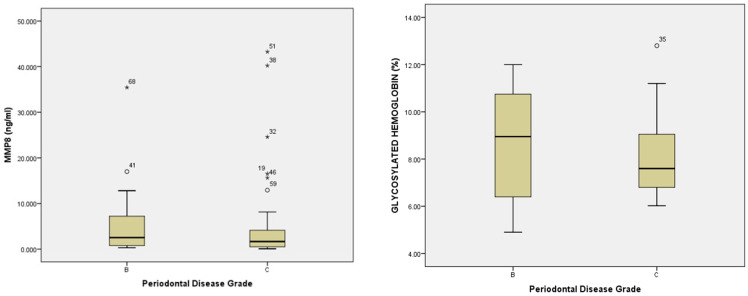
Distribution of Inflammatory and Glycemic Markers According to Periodontal Disease Grade (*n* = 79).

To complement the correlation analyses presented above, the distribution of inflammatory markers by periodontal disease grade was further explored, as detailed below and illustrated in Figure 2.

To complement the correlation analyses, additional visual exploration was conducted to evaluate how inflammatory markers vary according to periodontal disease severity. Boxplots were generated to illustrate the distribution of salivary IL-1β, IL-6, and MMP-8 levels across the two diagnostic grades observed in the study cohort: Grade B (moderate progression) and Grade C (rapid progression).

Although independent *t*-tests did not reveal statistically significant differences in marker concentrations between the two grades, the boxplots reveal informative trends regarding variability and central tendency. For instance, MMP-8 levels exhibited a wider range and more pronounced outliers in patients with Grade C periodontitis, suggesting greater heterogeneity in inflammatory response among those with rapid disease progression. A similar pattern was noted for IL-6, where values appeared more dispersed in Grade C, despite overlapping interquartile ranges. In contrast, IL-1β values showed relatively similar distributions between the two groups, with no major visual differences in medians or spread.

These graphical representations offer a nuanced perspective on the inflammatory burden associated with different stages of periodontal progression. They suggest that although mean values may not differ significantly, individual variability in the inflammatory profile could be greater among patients with more aggressive disease forms. This highlights the potential value of using salivary biomarkers not only for assessing average group trends but also for identifying high-risk individuals within broader diagnostic categories.

### 3.4. Correlations Between Self-Perceived Oral Health, Clinical Periodontal Parameters, and Disease Classification

A series of Pearson correlations was conducted to explore associations between self-reported oral health, clinical periodontal indicators, and periodontal disease classification. The variable self-assessment oral status was derived as a composite score based on patients’ responses to gum bleeding, tooth or gum sensitivity, and perceived tooth mobility. Similarly, self-assessment oral knowledge was computed from items related to patient–practitioner communication and perceived understanding of the link between diabetes and oral health.

A significant positive correlation was found between self-assessed oral knowledge and bleeding on probing (r = 0.28, *p* < 0.05), as well as with the plaque index (r = 0.23, *p* < 0.05), both showing small effect sizes (r^2^ = 0.08 and 0.05, respectively). These results suggest that increased awareness may coincide with higher levels of clinical inflammation and plaque, possibly reflecting patient recognition of ongoing periodontal problems.

Bleeding on probing also showed a strong correlation with plaque index (r = 0.82, *p* < 0.01), indicating a substantial overlap between biofilm accumulation and gingival inflammation (r^2^ = 0.60). Additionally, bleeding percentage correlated positively with probing depth (r = 0.22, *p* < 0.05) and CAL (r = 0.41, *p* < 0.01), with small to moderate effect sizes.

The plaque index was also positively correlated with CAL (r = 0.25, *p* < 0.01), reinforcing the role of plaque in tissue breakdown. Moreover, probing depth was significantly associated with the diagnostic grade of periodontitis (r = 0.25, *p* < 0.05), and a significant correlation was observed between disease stage and grade (r = 0.34, *p* < 0.01), reflecting internal consistency in the classification system.

**Figure 3:**
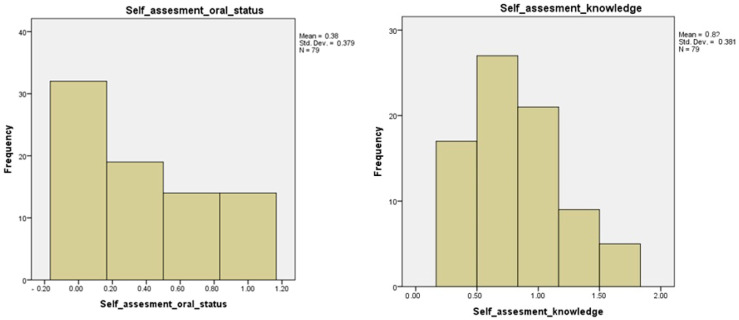
Distribution of self-perceived oral health symptoms and knowledge among diabetic patients (*n* = 79).

These findings support the complex interrelation between patients’ subjective perception of oral health and objective periodontal outcomes. Additionally, the alignment between diagnostic criteria and clinical measures further contextualizes these findings. The distributions of the composite scores for self-assessed oral status and oral health knowledge are illustrated in Figure 3. These visual representations help clarify the variability in patients’ subjective perceptions, revealing skewed distributions that suggest a general underreporting of oral symptoms and a limited understanding of the systemic–oral health connection.

### 3.5. Differences in Clinical and Inflammatory Parameters Based on Oral Health Self-Perception and Gender

To further explore how subjective perceptions of oral health align with objective clinical and biochemical parameters, a series of independent-samples *t*-tests were conducted. The dependent variables included self-assessment of oral status and self-assessment of oral health knowledge, both categorized using the mean value as a cutoff (M = 0.38 for oral status; M = 0.82 for knowledge). Gender was also tested as a grouping variable in relation to several periodontal and metabolic measures.

For self-assessment of oral status, no statistically significant differences were identified between the high and low scoring groups with respect to periodontal diagnosis (stage or grade), plaque index, bleeding on probing, IL-6, IL-1β, MMP-8, glycosylated hemoglobin (HbA1c), or diabetes duration. This suggests that perceived symptom severity was not systematically associated with clinical or inflammatory parameters.

In contrast, self-assessment of oral health knowledge yielded significant differences. Participants with above-average scores on this scale demonstrated significantly higher plaque levels (M = 40.21) compared to those scoring below average (M = 24.66), t (77) = 2.35, *p* < 0.05, with a moderate effect size (Cohen’s d = 0.53). Similarly, bleeding on probing was significantly elevated in the higher-knowledge group (M = 47.11) versus the lower-knowledge group (M = 30.26), t (77) = 2.39, *p* < 0.05, Cohen’s d = 0.54. These findings may reflect a form of reverse association, where individuals with greater awareness also exhibit more advanced disease signs, potentially due to heightened recognition of symptoms.

Finally, when comparing male and female participants, no significant differences were observed for any of the following: time since diabetes diagnosis, plaque percentage, bleeding percentage, inflammatory marker concentrations (IL-6, IL-1β, MMP-8), periodontal diagnosis (stage or grade), or either of the self-assessment measures. This indicates a relatively homogeneous distribution of clinical and subjective variables across gender lines in this sample (Table 4).

## 4. Discussion

This cross-sectional study aimed to evaluate the periodontal status of adult patients with type 1 and type 2 diabetes mellitus attending an outpatient diabetes care facility in Timișoara, Romania. Additionally, it sought to identify factors influencing the association between periodontitis and glycemic control. While our previous studies conducted within the same patient population examined subjective perceptions of oral health and behavioral patterns among diabetic individuals [3,9], the present research builds upon these findings by integrating objective clinical parameters, inflammatory biomarkers, and validated periodontal indices. This multi-level approach offers a deeper insight into the biological and behavioral interface underlying the diabetes–periodontitis link. A comprehensive full-mouth periodontal examination was conducted, utilizing standardized classification criteria, which are widely recommended for investigating the systemic implications of periodontal disease [25].

A comprehensive patient history and clinical evaluation are essential. In type I diabetes mellitus (DM), symptoms typically present abruptly over the course of days or weeks. Conversely, in type II DM, clinical signs such as fatigue, numbness, pruritus, drowsiness, increased thirst, polyuria, and depressive symptoms tend to develop gradually and may be obscured by factors such as aging or obesity. Intraorally, there are no specific lesions or periodontal patterns directly attributed to hyperglycemia. However, individuals with diabetes exhibit a higher prevalence and greater severity of periodontitis. Moreover, poor glycemic control has been associated with manifestations such as xerostomia, burning mouth sensation, coated and fissured tongue, oral candidiasis, increased caries risk, and delayed wound healing [26]. Individuals presenting with diabetes-related symptoms or those who are overweight should be referred to a physician for appropriate laboratory evaluations, such as fasting plasma glucose measurement. Monitoring of glycosylated hemoglobin (HbA1c) is recommended every three months, corresponding to the approximate 120-day lifespan of red blood cells. HbA1c levels provide an estimate of average blood glucose concentrations over the preceding three months. In healthy individuals, HbA1c values are typically around 5%, and even in patients with diabetes, levels should ideally remain below 7.0–7.5% [27,28].

In our study, higher levels of self-reported oral health knowledge were associated with increased plaque accumulation and bleeding on probing, potentially reflecting heightened symptom awareness rather than effective preventive behaviors. This observation highlights a possible disconnect between knowledge and practical implementation, emphasizing the need for structured behavioral interventions and motivational interviewing strategies within diabetes care frameworks. Recent findings from a Romanian self-report study [29] on diabetic and non-diabetic individuals revealed significant associations between perceived periodontal symptoms (such as gingival bleeding, halitosis, and tooth mobility) and modifiable behaviors, including oral hygiene habits, diet, and stress levels. Notably, diabetic patients were more likely to report gingival symptoms and dry mouth, but paradoxically less likely to use interdental hygiene aids.

Previous studies on diabetes mellitus have reported highly variable prevalence rates of periodontitis, ranging from 13.6% to 97.7%, likely due to differences in ethnic backgrounds and the criteria used to define periodontal disease severity [30,31,32,33]. In an epidemiological survey of our Caucasian population, 52% of adults over the age of 60, regardless of their diabetes status, were found to have severe periodontitis based on CDC/AAP classification criteria. Moreover, the overall prevalence of periodontitis in our study reached 91%, with 63.4% classified as severe cases, highlighting the critical need for timely intervention, improved treatment strategies, and enhanced preventive oral care programs [34].

Several biological mechanisms have been proposed to explain the impact of periodontitis on metabolic control, though the supporting evidence remains moderate. Local periodontal inflammation creates an ulcerated interface that may facilitate the entry of bacteria and inflammatory mediators into the bloodstream, potentially influencing systemic health and exacerbating metabolic dysregulation [19,35,36,37].

Both diabetes and periodontal disease are significant public health concerns, exerting a profound impact on patients’ quality of life and overall health outcomes. Research exploring the interplay between these two conditions remains fragmented, though current evidence suggests a bidirectional relationship. This connection is largely attributed to shared inflammatory pathways, including bacteremia, cytokine production, oxidative stress, and endothelial dysfunction.

The bidirectional relationship between periodontal disease and diabetes is well-established, with immune system dysfunction playing a key role in the pathophysiological mechanisms linking the two conditions. However, despite its clinical significance, this association remains under-recognized [38,39]. Research indicates that non-surgical periodontal treatment can contribute to an approximate 0.4% reduction in glycated hemoglobin (HbA1c) levels, highlighting its potential role in glycemic management [40,41,42]. The concept of periodontitis as the “sixth complication of diabetes” was introduced by Löe in 1993 [43], based on findings that individuals with type 2 diabetes have a threefold increased risk of developing periodontal disease compared to non-diabetic individuals [40,44]. Furthermore, severe periodontitis is closely linked to poorly controlled diabetes, as chronic hyperglycemia promotes the release of inflammatory mediators that exacerbate periodontal tissue destruction [45,46].

Experimental and observational studies have significantly advanced our understanding of the intricate connection between diabetes and periodontitis. Evidence suggests that non-surgical periodontal therapy can positively influence glycemic control, although its effectiveness depends on multiple factors [47,48]. Further research is needed to explore the mechanisms underlying this relationship and optimize periodontal treatment strategies for individuals with diabetes. Given that previous studies [49,50] have demonstrated the effectiveness of periodontal therapy in reducing HbA1c levels, we hypothesize that periodontal health may have a negative impact on diabetes management. Therefore, we propose that periodontal treatment could contribute to improved metabolic control in patients with type 2 diabetes.

Interleukins (ILs), a diverse family of over 40 cytokines [51], play a pivotal role in orchestrating immune and inflammatory responses. Their dysregulation has been increasingly implicated in the pathophysiology of both diabetes mellitus and periodontal disease. In hyperglycemic environments, particularly within pancreatic islets, elevated glucose levels can induce the secretion of pro-inflammatory interleukins such as IL-1β, contributing to β-cell dysfunction and apoptosis through oxidative stress and inflammatory signaling. Similarly, in periodontal tissues, ILs—especially IL-1β and IL-6—are key mediators of local tissue destruction, promoting osteoclastogenesis and extracellular matrix degradation. Recent evidence suggests that interleukin-targeted therapies, such as IL-1 receptor antagonists, may not only modulate systemic inflammation but also improve glycemic control and β-cell secretory function. Given their dual role in metabolic and oral inflammatory pathways, further exploration of specific IL profiles may enhance our understanding of the shared pathogenesis linking diabetes and periodontitis, and guide the development of novel, integrative therapeutic approaches [52].

The results of this study are consistent with recent research highlighting the central role of inflammatory mediators in the pathogenesis of periodontal disease in diabetic patients. For example, a recent study demonstrated that elevated levels of IL-1β, IL-6, and MMP-8 are associated with increased periodontal disease severity among diabetic patients, suggesting a bidirectional relationship between glycemic control and periodontal inflammation [1]. These findings reinforce the hypothesis that periodontal interventions may have a positive impact on glycemic control and reduce the systemic inflammatory response [9].

Elevated levels of serum reactive oxygen species, interleukin (IL)-1, IL-6, tumor necrosis factor (TNF)-α, and C-reactive protein (CRP) have been observed in patients with established type 2 diabetes mellitus (T2DM), indicating their potential role in periodontal tissue destruction. This suggests that as chronic hyperglycemia persists and worsens over time, the inflammatory response in periodontal tissues is likely to intensify, contributing to disease progression and increased tissue breakdown [53]. The modest correlation between IL-6 and HbA1c in the present study may reflect the localized periodontal expression of inflammatory mediators, rather than systemic spillover. Alternatively, it suggests that IL-6′s contribution to insulin resistance is context-dependent, influenced by factors such as adiposity, hepatic function, and overall inflammatory burden. This could explain why the observed association between glycemic control and periodontal inflammation was weak, implying that local inflammatory processes may progress independently of systemic metabolic regulation, while still contributing cumulatively to the broader inflammatory load in diabetic patients.

Several studies have established a link between matrix metalloproteinases (MMPs) and periodontitis [54], with MMP-8 being the predominant collagenase (80%) detected and analyzed in gingival tissue and gingival crevicular fluid (GCF) [55]. Periodontal bacteria stimulate immune cells, triggering the production and release of MMP-8 into the bloodstream, leading to elevated serum MMP-8 levels in patients with generalized and aggressive periodontitis. A recent literature review [11] emphasized the increasingly recognized bidirectional relationship between diabetes mellitus and periodontal disease, with shared inflammatory and immunological pathways contributing to disease progression in both directions. Hyperglycemia promotes elevated local levels of IL-1β, TNF-α, and MMP-9, which not only exacerbate periodontal tissue destruction but also contribute to systemic insulin resistance and β-cell apoptosis. Furthermore, the presence of diabetes has been shown to alter the composition and function of the oral microbiome, favoring dysbiosis and the persistence of inflammophilic microbial species. Emerging evidence suggests that these microbiome alterations may persist even in clinically healthy individuals with diabetes, highlighting a latent risk for periodontal deterioration. These insights reinforce the importance of comprehensive management strategies that address both glycemic control and periodontal inflammation, potentially through adjunctive therapies such as anti-cytokine agents or microbiota-modulating interventions.

Given the bidirectional interplay and emerging data on the systemic benefits of periodontal therapy, integrating dental assessments into diabetes management protocols—especially in primary care settings—could yield meaningful improvements in both oral and metabolic outcomes. Altogether, the findings highlight the importance of recognizing periodontitis not just as an oral issue, but as a systemic contributor to overall health in diabetic patients. Incorporating dental care into diabetes management, supported by targeted health education, may offer significant benefits in reducing inflammatory load and improving long-term outcomes.

However, certain limitations must be acknowledged. A longer follow-up period could provide a more comprehensive understanding of the long-term effects of periodontal therapy on glycemic control. Additionally, natural history studies suggest that changes in HbA1c levels are influenced by multiple factors, making it challenging to isolate the specific impact of periodontal treatment. The potential role of periodontal therapy in modulating HbA1c levels remains a subject of debate, warranting further investigation. Moreover, the cross-sectional design of this study prevents the establishment of a cause-and-effect relationship, limiting the strength of the conclusions. The sample size was also restricted due to specific enrollment criteria, which may affect the generalizability of the findings. Future longitudinal studies with larger cohorts are necessary to validate these associations and better understand the interplay between periodontal health and metabolic control in diabetic patients.

Recent literature provides compelling evidence supporting the interplay between oral inflammatory conditions and systemic immune responses, which may further illuminate the findings of this review. For instance, Miller et al. (2021) [21] demonstrated that salivary biomarkers such as interleukin-1β (IL-1β), interleukin-6 (IL-6), and matrix metalloproteinase-8 (MMP-8) are significantly elevated in patients with type 2 diabetes mellitus and are associated with periodontal inflammation. These biomarkers reflect underlying cytokine dysregulation and reinforce the notion of a bidirectional relationship between systemic metabolic imbalance and oral health status. While this study focused on diabetic patients, its implications can extend to pregnancy [22]—a physiological state characterized by hormonal and immunological fluctuations—which may similarly predispose individuals to periodontal disease via altered immune-inflammatory pathways.

Additionally, Guerrero-Gironés et al. (2021) [56] conducted a systematic review exploring the association between pulpal-periapical pathology and autoimmune diseases, such as type 1 diabetes, rheumatoid arthritis, and inflammatory bowel disease. Their findings suggest that immune-mediated systemic inflammation may negatively impact periapical healing and increase susceptibility to endodontic pathology. Considering that pregnancy involves temporary immune modulation, often shifting toward a pro-inflammatory profile in certain trimesters, it is plausible that similar mechanisms may be at play, contributing to increased periodontal and periapical vulnerability. These perspectives advocate for the integration of salivary biomarker monitoring and a more comprehensive systemic approach to oral healthcare in pregnant patients, promoting earlier diagnosis and targeted interventions.

### Future Perspectives

The results of this study support several future directions in both research and clinical practice. To better understand the temporal and potentially causal relationships between glycemic control, salivary inflammatory biomarkers, and periodontal disease severity, larger-scale longitudinal studies are recommended. Future investigations should focus on validating standardized cutoff values for key biomarkers such as IL-1β, IL-6, and MMP-8, and exploring their potential for monitoring periodontal status in diabetic populations. Incorporating salivary biomarker assessment into routine diabetes care protocols could facilitate earlier identification of periodontal inflammation and guide individualized prevention strategies. These advancements will benefit from strengthened collaboration between dental care providers and diabetologists, fostering a more integrated model of care that addresses the systemic–oral health interface in diabetic patients.

## 5. Conclusions

This study confirms the clinically relevant association between diabetes mellitus and periodontitis, emphasizing the role of inflammation as a shared pathological mechanism. The high prevalence of advanced periodontal disease and the elevated inflammatory indices in diabetic patients underline the need for integrating periodontal screening into routine diabetes care. Significant correlations between salivary biomarkers (IL-1β, IL-6, MMP-8) and periodontal parameters, particularly bleeding and plaque index, support the involvement of a localized inflammatory response that may operate independently from glycemic control. Interestingly, increased oral health knowledge did not align with better clinical outcomes, suggesting a disconnect between awareness and behavior. The low frequency of patient–provider communication on oral health highlights a need for improved interdisciplinary collaboration and patient education. Overall, these findings support the inclusion of periodontal evaluation and education in diabetes management protocols, especially at the primary care level, to reduce the inflammatory burden and potentially improve systemic outcomes.

## Figures and Tables

**Table 1:** Demographic characteristics of the study population: Gender, type of diabetes mellitus, and duration since diagnosis.

Variable	N (%)
Gender	
Male	35 (44.3%)
Female	44 (55.7%)
Type of Diabetes Mellitus	
Type 1	11 (13.9%)
Type 2	68 (86.1%)
Duration of Diabetes	
<1 year	15 (19%)
1–5 years	18 (22.8%)
6–10 years	13 (16.5%)
10–14 years	11 (13.9%)
15–19 years	9 (11.4%)
20–24 years	8 (10.1%)
>29 years	5 (6.3%)

**Table 2:** Clinical periodontal parameters and distribution of periodontitis stage and grade in diabetic patients.

Parameter	Category/Value	Frequency (*n*)	Percentage (%)
Probing Pocket Depth (mm)	Mean ± SD	4.00 ± 0.69	
Clinical Attachment Level (mm)	Mean ± SD	−2.59 ± 4.50	
Plaque Index	Mean ± SD (%)	45.73 ± 20.27	
Bleeding on Probing	Mean ± SD (%)	55.14 ± 18.93	
Periodontal Disease Stage	Stage III	27	34.2%
	Stage IV	52	65.8%
Periodontal Disease Grade	Grade B (moderate rate)	22	27.8%
	Grade C (rapid rate)	57	72.2%

**Table 3:** Statistically significant correlations between salivary inflammatory markers and clinical periodontal parameters.

	IL-1β	IL-6	MMP 8	Periodontal Stage	Bleeding (%)	Plaque Index (%)
IL-1β		0.34	−0.24	−0.23		
IL-6	0.34			−0.33		
MMP-8	−0.24				0.26	0.28
Periodontal Stage	−0.23	−0.33				
Bleeding (%)			0.26			
Plaque Index (%)			0.28			

**Table 4:** Gender-based differences in clinical, biochemical, and self-reported oral health parameters among diabetic patients.

Variables Gender		N	Mean	Std. Deviation
Duration of Diabetes (years)	M	35	3.00	1.49
	F	44	3.56	2.08
Plaque Index (%)	M	35	30.12	30.37
	F	44	32.69	29.97
Bleeding on Probing (%)	M	35	36.92	31.86
	F	44	38.36	32.38
Salivary MMP-8 (ng/mL)	M	35	6.27	11.12
	F	44	5.02	7.70
Glycated Hemoglobin (HbA1c, %)	M	25	8.09	1.91
	F	35	8.32	1.74
Salivary IL-6 (pg/mL)	M	35	24.20	29.03
	F	44	45.01	56.53
Salivary IL-1β (pg/mL)	M	35	782.02	651.45
	F	44	888.08	740.14
Periodontal Disease Grade	M	35	1.71	0.45
	F	44	1.72	0.45
Periodontal Disease Stage	M	35	3.71	0.45
	F	44	3.59	0.54
Self-Assessed Oral Health Knowledge Score	M	35	0.90	0.36
	F	44	0.75	0.38

## Data Availability

The data presented in this study are available on request from the corresponding author.

## Abbreviations

The following abbreviations are used in this manuscript:

**DM** I	Diabetes Mellitus
**PD**	Periodontal Disease
**T1DM**	Type 1 Diabetes Mellitus
**T2DM**	Type 2 Diabetes Mellitus
**PPD**	Probing Pocket Depth
**CAL**	Clinical Attachment Level
**PI**	Plaque Index
**BO**	Bleeding on Probing
**IL-1β**	Interleukin-1 beta
**IL-6**	Interleukin-6
**MMP-8**	Matrix Metalloproteinase-8
**HbA1c**	Glycated Hemoglobin
**ELISA**	Enzyme-Linked Immunosorbent Assay
**CPITN**	Community Periodontal Index of Treatment Needs
